# How to use CT texture analysis for prognostication of non-small cell lung cancer

**DOI:** 10.1186/s40644-016-0065-5

**Published:** 2016-04-11

**Authors:** Kenneth A. Miles

**Affiliations:** Department of diagnostic imaging, Princess Alexandra Hospital, Woolloongabba, Queensland, Australia; Institute of Nuclear Medicine, University College London, Euston Road, London, UK

## Abstract

Patients with non-small cell lung cancer frequently demonstrate differing clinical courses, even when they express the same tumour stage. Additional markers of prognostic significance could allow further stratification of treatment for these patients. By generating quantitative information about tumour heterogeneity as reflected by the distribution of pixel values within the tumour, CT texture analysis (CTTA) can provide prognostic information for patients with NSCLC. In addition to describing the practical application of CTTA to NSCLC, this article discusses a range of issues that need to be addressed when CTTA is included as part of routine clinical care as opposed to its use in a research setting. The use of quantitative imaging to provide prognostic information is a new and exciting development within cancer imaging that can expand the imaging specialist’s existing role in tumour evaluation. Derivation of prognostic information through the application of image processing techniques such as CTTA, to images acquired as part of routine care can help imaging specialists make best use of the technologies they deploy for the benefit of patients with cancer.

## Background

**Fig. 1:**
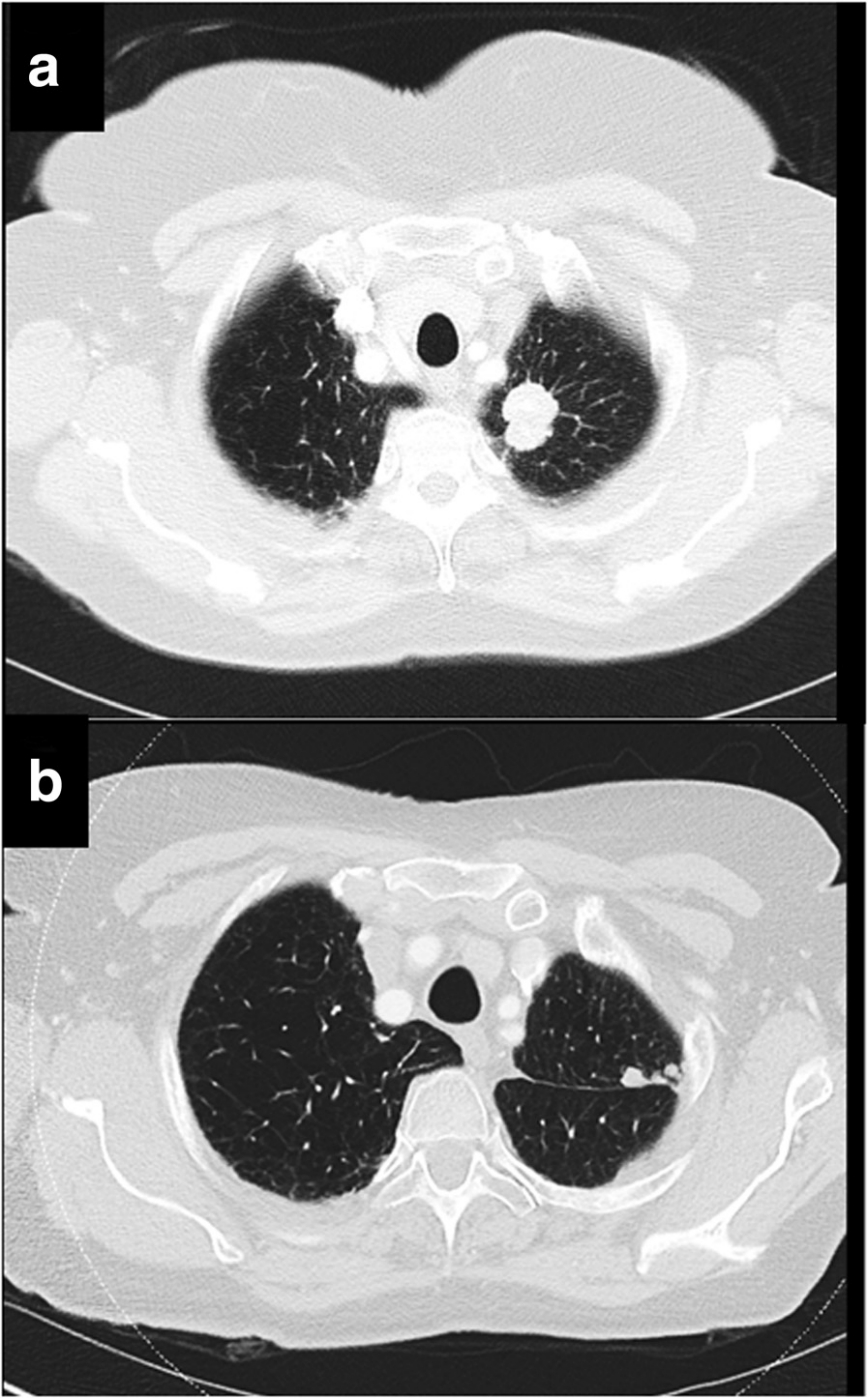
The potential for prognostic biomarkers to stratify care for patients with NSCLC. CT showing left upper lobe NSCLC at initial staging **a**. Based on current practice, the patient underwent surgery without adjuvant chemotherapy. CT performed 25 months later shows local recurrence **b**. A biomarker deployed at staging may have categorised the patient as high-risk for recurrence, implying a potential benefit from adjuvant chemotherapy

Lung cancer remains the leading cause of cancer death in Western societies, with more than 75 % of cases comprising non-small cell lung cancer (NSCLC). Tumour stage is the most important prognostic variable for survival, and this parameter makes a major contribution to clinical decisions concerning the benefits of surgery, chemotherapy and/or radiotherapy for individual patients. However, patients with the same tumour stage frequently demonstrate differing clinical courses. Hence, there is a need for additional markers of prognostic significance that could for example, identify those patients with the highest likelihood of post-surgical recurrence who might benefit most from adjuvant chemotherapy (Fig. 1), or alternatively to recognise those patients with advanced disease who are unlikely to get sufficient survival benefit to justify the morbidity of chemotherapy in a palliative setting.

Computed Tomography (CT) plays a key role in staging of NSCLC, either as a stand-alone technique or during Positron Emission Tomography, and the use of CT to provide additional prognostic markers can form a natural extension to this role. Deriving markers of prognosis from existing CT images avoids the cost and radiation exposure associated with techniques such as perfusion CT that require specialised data acquisitions. CT texture analysis (CTTA) is an image processing method that can be applied to routinely acquired images to provide additional quantitative information about tumour heterogeneity as reflected by the distribution of pixel values within the tumour. Tumour heterogeneity is an important biological characteristic related to tumour aggression and response to treatment. There is an increasing body of evidence demonstrating the ability of CTTA to provide prognostic information for patients with NSCLC and other tumours [1–5].

### Integration of CTTA into clinical workflow

**Fig. 2:**
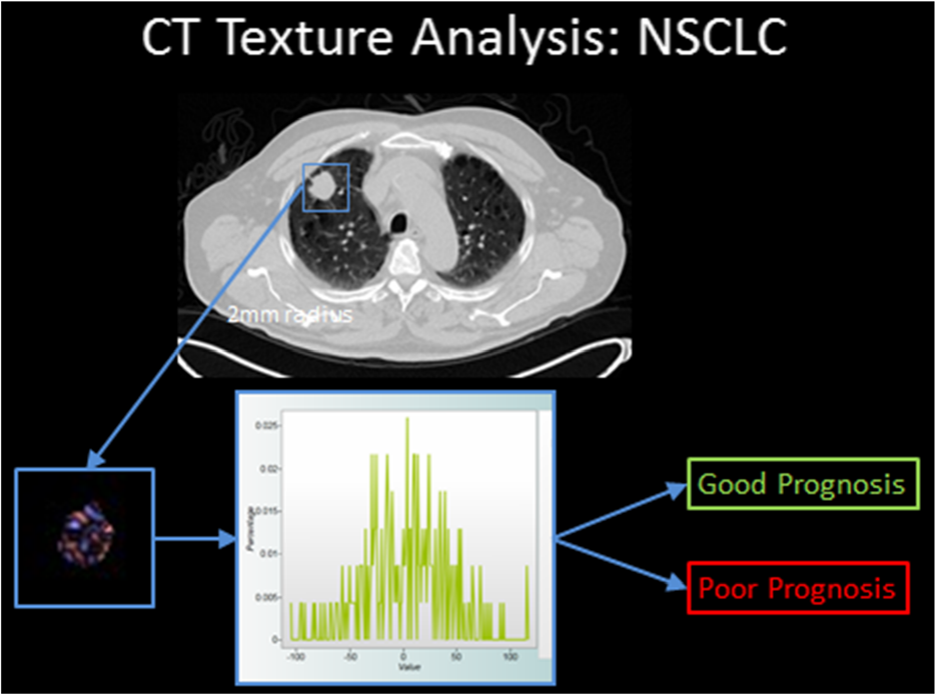
Summary of the filtration-histogram method for CTTA. The conventional CT image (top) is filtered to highlight objects of a pre-selected size. The distribution of tumour features within the filtered image as assessed using standard statistical parameters derived from the corresponding histogram, provides an indication of prognosis

At present, none of the currently available image viewing or Picture Archiving and Communication software packages has integrated CTTA into their products. A stand-alone software that implements the filtration/histogram method is available commercially (Fig. 2) but needs to be interfaced with the software used for routine radiological diagnosis. A DICOM export function is a suitable and commonly available option but other arrangements are feasible. Because manual segmentation from the mediastinum, chest wall or adjacent consolidated lung is required for analysis for some tumours, CTTA is best performed by the reporting radiologist. Therefore, CTTA software needs to be installed on the same workstation as that used for routine image review or available on a separate but immediately adjacent workstation. This arrangement also allows the results of CTTA to be included within the conventional report of the images undergoing analysis, ensuring clinical immediacy and relevance. An ability to display CTTA results at the multi-disciplinary meeting at which treatment decisions are made enables CTTA to be integrated with other clinical and pathological information. At our institution, the lung cancer registry software has been modified to incorporate CTTA results.

### Image selection

Although any CT image can in principle be analysed using CTTA, at our institution we currently restrict analysis to the low-dose CT (LDCT) component of Positron Emission Tomography (PET)/CT examinations for the following reasons: Firstly, the prognostic value of CTTA in NSCLC has been more extensively clinically validated for LDCT than for diagnostic CT images, including derivation and testing of cut-off values from separate patient cohorts [6]. Secondly, CTTA results can be affected by reconstruction parameters [7] which are more likely to be varied in clinical routine for diagnostic CT than LDCT. Thirdly, the PET images can be useful in guiding the delineation of tumour margins, particularly when adjacent to pulmonary consolidation. Finally, the derivation and reporting of quantitative image biomarkers requires a critical approach to image analysis that is often more established within nuclear medicine.

### Region of interest construction

**Fig. 3:**
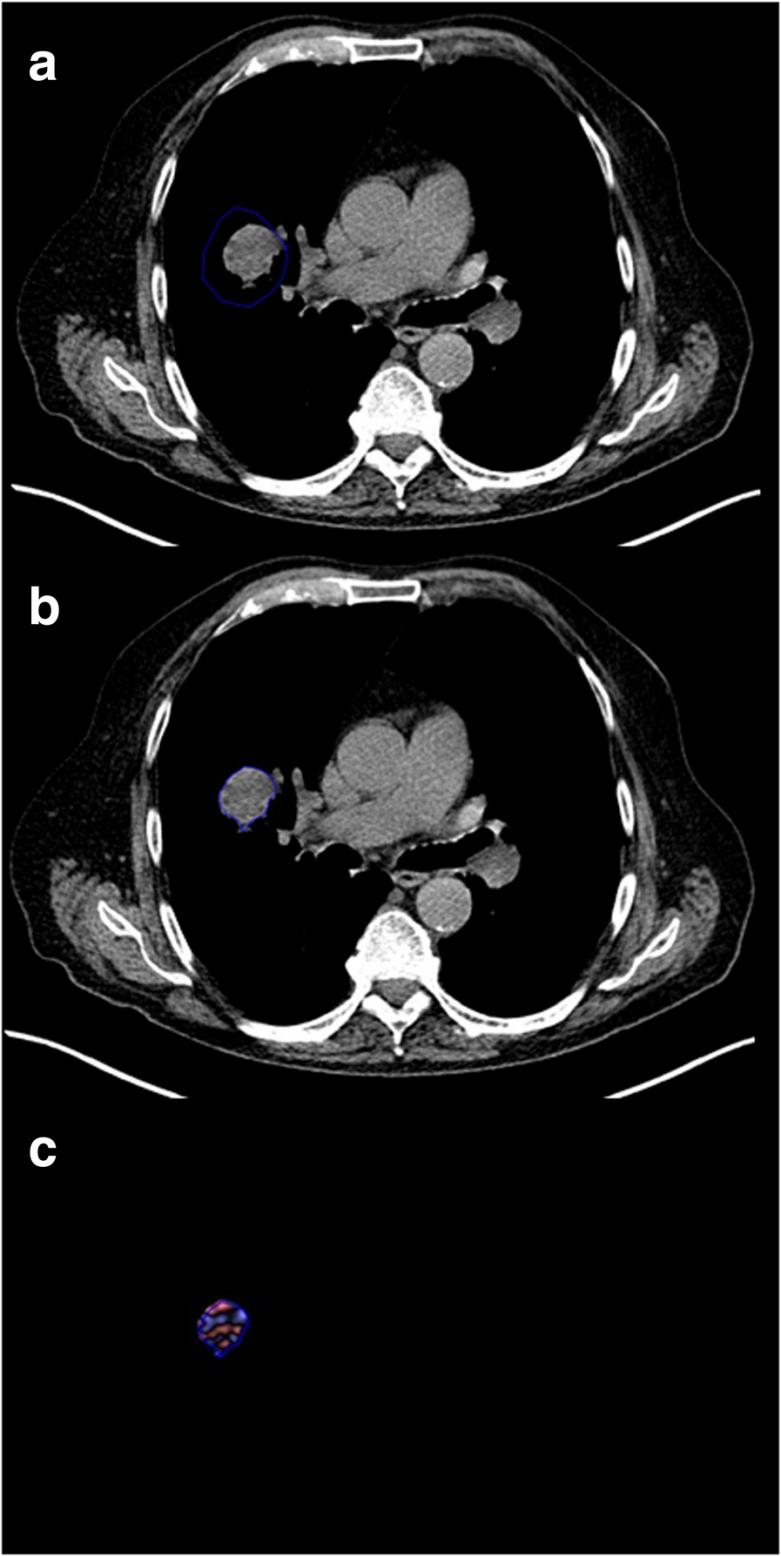
When using automatic segmentation for tumour Regions of Interest (ROIs), the initial manually constructed ROI (**a**) can include surrounding lung. The segmentation software then redifines the ROI to exclude lung tissue **b**. The filtered tumour image (**c**) is used for derivation of texture parameters by histogram analysis

**Fig. 4:**
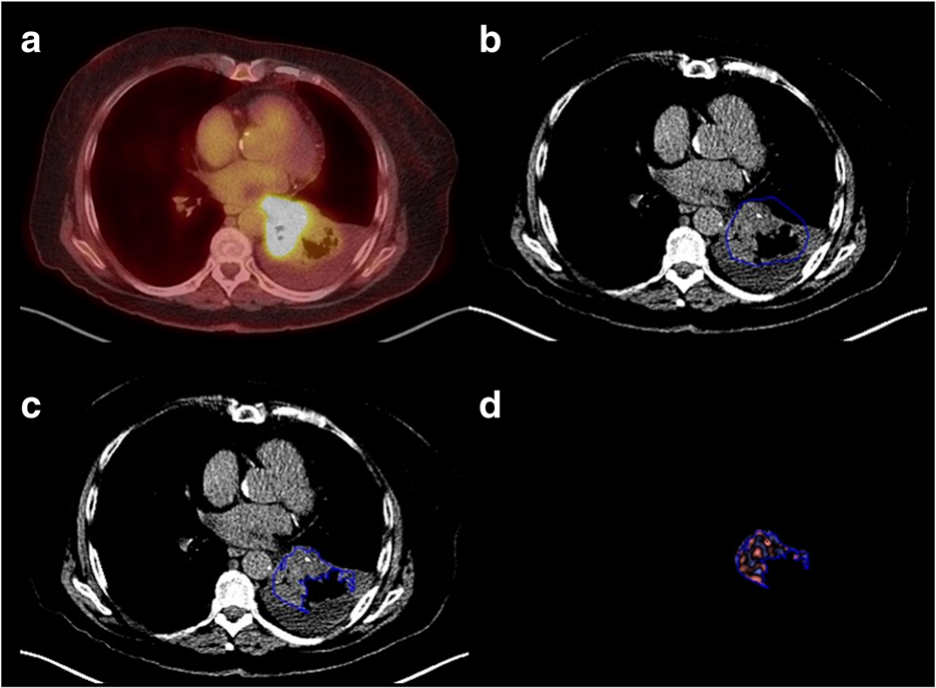
Left lower lobe NSCLC showing cavitation and adjacent consolidation. The fused FDG-PET/CT image (**a**) and narrow CT windows (**b**) can assist identification of the tumour margins. Using automatic segmentation, the initial manually constructed ROI (**b**) includes adjacent lung and the area of cavitation but excludes the adjacent mediastinal structures and pulmonary consolidation. The final ROI defined by the automated segmentation procedure (**c**) exlcudes the adjacent lung and area of cavitation. The final filtered tumour image is shown in (**d**)

**Fig. 5:**
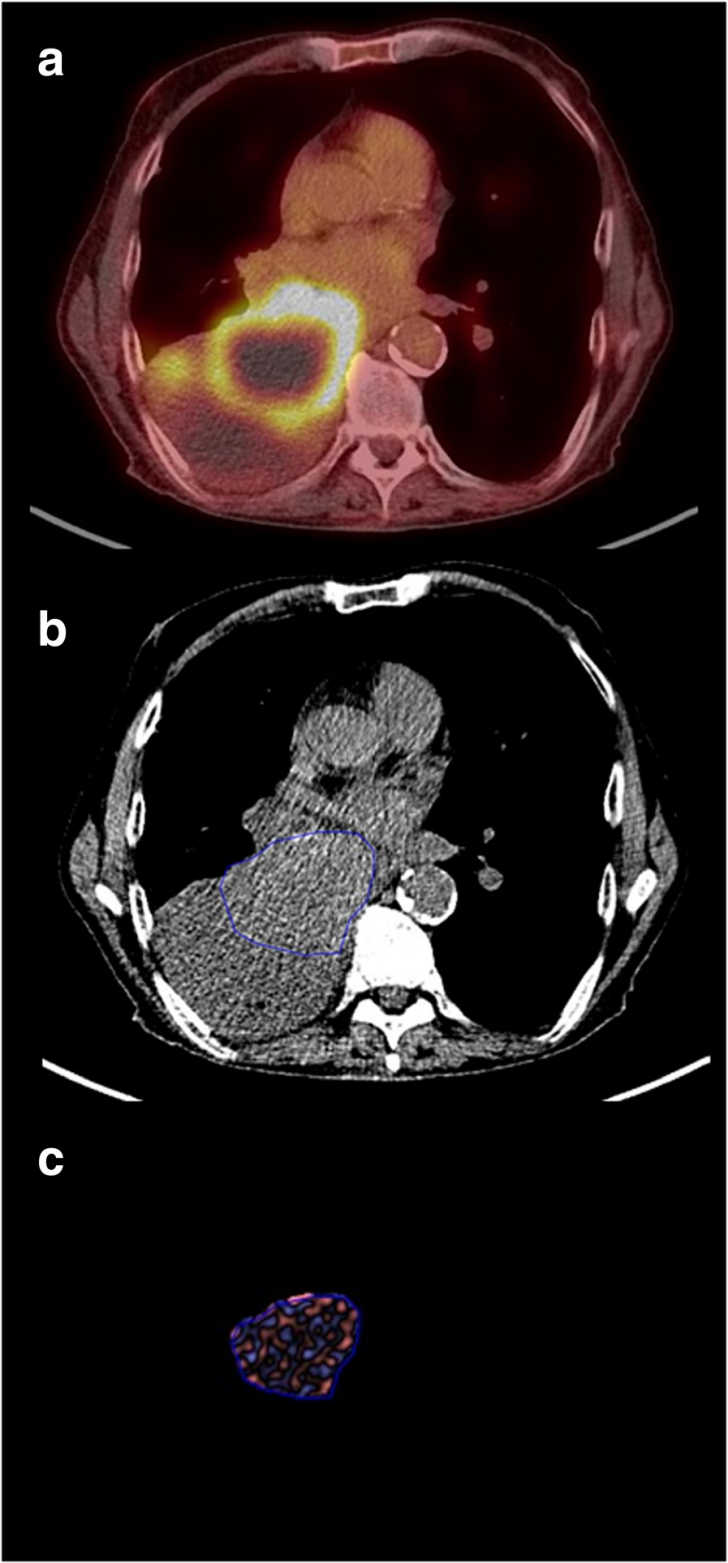
Right lower lobe NSCLC showing necrosis without cavitation (photopaenia on FDG-PET/CT) and adjacent pulmonary consolidation (**a**). Due to minimal contact with aerated lung, the tumour ROI has been constructed manually (**b**), using the fused PET/CT image and narrow windows for guidance. The area of necrosis without cavitation is included in the the ROI and the final filtered tumour image (**c**)

The single CT slice that displays the largest cross-section of the tumour is selected for analysis and displayed in soft-tissue windows. When constructing the tumour region of interest (ROI), automated segmentation procedures should be used wherever possible to optimise consistency in analysis between operators. Computer automated segmentation of tumour relative to aerated lung is straightforward. When a tumour is completely surrounded by aerated lung, segmentation tools allow the operator to construct a ROI beyond the tumour edge within which the precise tumour margins are defined by the software algorithm (Fig. 3). However, if the tumour is in contact with chest wall, mediastinum, pleural fluid or consolidated lung, the operator must manually define the tumour soft-tissue interface accurately, erring on the inside of the tumour. For the remaining borders where tumour is in contact with aerated lung, this section of ROI can be drawn within the lung (i.e. outside the tumour) leaving the segmentation algorithm to complete the definition of the tumour edge automatically. The use of narrow CT windows (e.g. level: 40HU, width 150HU) and reference to fused PET/CT images can assist the definition of tumour boundaries (Figs. 4 and 5). Areas of tumour cavitation visible on CT should not be included within the ROI but may be excluded by segmentation tools (Fig. 4). Areas that are necrotic on FDG-PET (seen as central photopaenia) but exhibit soft-tissue density on CT should be included within the ROI as there is currently no data available to indicate the likely impact of excluding such areas on the derived CTTA values (Fig. 5).

### Reporting

CTTA software typically returns a range of texture parameters for the constructed tumour ROI. For the filtration-histogram CTTA approach, these parameters characterise the histogram of pixel intensity values within the ROI for a series of filtered images highlighting features of a specified size. Each parameter has a different relationship with the size, number, brightness and variability of features in the original CT image [8]. A choice needs to be made as to which of these parameters should be included in the final report along with their respective cut-off values defining good and poor prognosis. This choice can be based on previously published reports, ideally confirmed by a retrospective analysis of a local cohort of patients. On this basis, we currently report kurtosis and entropy values for filtered images highlighting objects of 4 mm radius, indicating that positive kurtosis and/or an entropy value of > 4.57 are associated with poorer survival.

### Quality assurance & audit

CTTA parameters reflect variations in x-ray attenuation (measured in Hounsfield Units) within the tumour. The accuracy of CT attenuation values is checked as part of the routine quality procedures recommended by the equipment manufacturers. Nevertheless, the potential sources of variability in CTTA values between different sites have not been fully characterised. It is therefore essential to audit the prognostic performance of results acquired locally before full implementation of CTTA as a biomarker in clinical practice. If the audit indicates that selection of different CTTA parameters and/or adjustment of cut-off values is required to optimise prognostic performance, new cut-off values should be determined from one patient cohort and tested in another. Alternatively, a cross-validation procedure may be used [6]. Auditing of CTTA results is also required after change of CT X-ray tube or installation of a new system.

### Clinical engagement

**Fig. 6:**
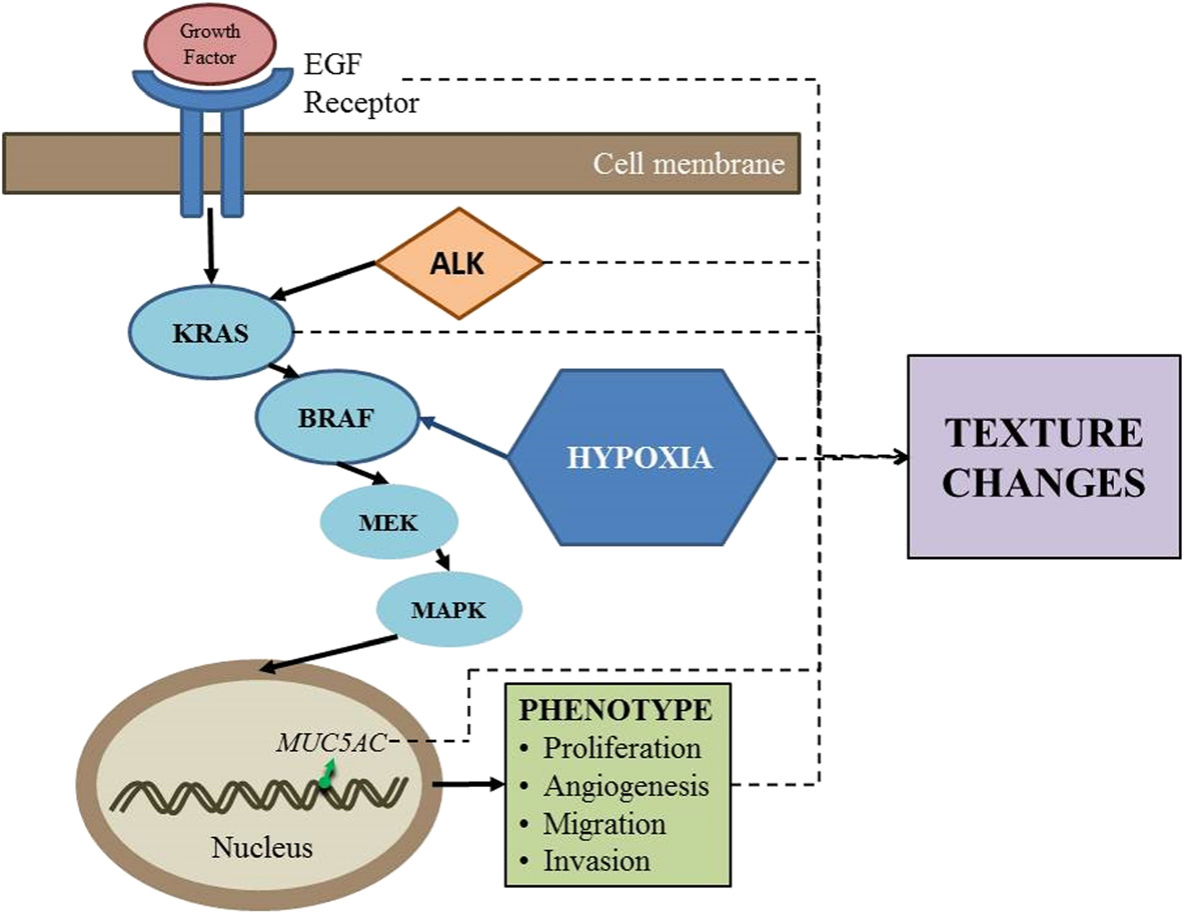
MAPK pathway and CTTA in NSCLC. Dotted lines indicate correlations between MAPK biology and CTTA in NSCLC demonstrated through clinical research

Acceptance of prognostic imaging biomarkers by clinicians is an essential pre-requisite to adoption into clinical practice and communication of a convincing case for the use of CTTA as a marker of prognosis is important for achieving clinical engagement. A “black box” approach which fails to enunciate the biological basis of the imaging biomarker values is unlikely to be successful even in the presence of compelling data linking the measurements to prognosis. In NSCLC, emerging evidence that points to the intra-tumour heterogeneity reflected by CTTA as being a phenotypic consequence of activation of the MAPK tumour pathway provides a clinically and therapeutic relevant foundation for CTTA as a prognostic indicator. Studies have shown CTTA values in NSCLC to be related to hypoxia, mutations in EGFR and KRAS genes, and ALK gene re-arrangements [3, 5, 9–12], all processes with links to the MAPK pathway which in turn, has an established relationship with survival (Fig. 6). Current works in progress has identified a correlation between kurtosis values in NSCLC and expression of the mucin production gene, Mucin5AC, which is considered a marker of MAPK pathway activation. As mucin produces lower attenuation of x-rays than soft-tissue, this association has disclosed a plausible connection between the MAPK pathway and CT signal in NSCLC.

## Conclusion

The use of quantitative imaging to provide prognostic information is a new and exciting development within cancer imaging that can expand the imaging specialist’s existing role in qualitative and semi-quantitative assessments of prognosis such as TNM staging and changes in tumour appearances during serial imaging. Parallel developments are occurring, or have already emerged, in other areas of imaging such as CT coronary calcium scoring for risk-assessment in patients with coronary artery disease. Cancer patients often need to undergo a range of investigations and there is a responsibility to maximise the clinically relevant information obtainable from these procedures, particularly when they entail ionising radiation. Derivation of prognostic information through the application of image processing techniques such as CTTA, to images acquired as part of routine care can help imaging specialists make best use of the technologies they deploy for the benefit of patients with cancer.

## Abbreviations

ALK: Anaplastic Lymphoma Receptor Tyrosine Kinase
BRAF: v-Raf murine sarcoma viral oncogene homolog B
CT: Computed Tomography
CTTA: computed tomography texture analysis
EGFR: Epidermal Growth Factor Receptor
KRAS: V-Ki-ras2 kirsten rat sarcoma viral oncogene homolog
LDCT: low-dose computed tomography
MAPK: mitogen activated protein kinase
MEK: MAPK/ERK kinase
NSCLC: non-small cell lung cancer
PET: Positron Emission Tomography
ROI: region of interest
